# Management of *Coxiella burnetii* Endocarditis in a Child With Congenital Heart Disease

**DOI:** 10.1155/crpe/8653626

**Published:** 2026-05-28

**Authors:** Emilija Zimnickaite, Andrea Dulcey, Christoph Aebi, Andrea Duppenthaler, Nina Schöbi, Fabienne Stoller, Florian Arndt, Philipp K. A. Agyeman

**Affiliations:** ^1^ Department of Pediatrics, Inselspital, University Hospital of Bern, University of Bern, Bern, Switzerland, unibe.ch; ^2^ Division of Pediatric Infectious Disease, Department of Pediatrics, Inselspital, University Hospital of Bern, University of Bern, Bern, Switzerland, unibe.ch; ^3^ Department of Cardiology, Center for Congenital Heart Disease, Inselspital, University Hospital of Bern, University of Bern, Bern, Switzerland, unibe.ch

**Keywords:** case report, child, congenital heart defect, *Coxiella burnetii*, infective endocarditis

## Abstract

A 6‐year‐old male patient with pulmonary atresia and recent valve replacement with a Melody valve presented with a 6‐week history of fever and cough. He was diagnosed with *Coxiella burnetii* endocarditis based on a *C. burnetii* Phase I IgG antibody titre of 1:65,536 and increased metabolic activity surrounding his prosthetic cardiac valve on positron emission tomography scan. Treatment was initiated with doxycycline and hydroxychloroquine. However, despite dose escalation, target plasma concentrations were not achieved. The patient experienced rapid clinical improvement, and serological markers declined gradually. Antimicrobial therapy was discontinued after 40 months, as predefined serological criteria were met, clinical course was favourable, and a follow‐up positron emission tomography scan demonstrated a significant reduction in metabolic activity around the prosthetic valve. Up to 23 months after cessation of therapy, the patient showed no evidence of active *C. burnetii* endocarditis during follow‐up. The Melody valve maintained its function, and valve replacement was not required. Clinicians should employ an individualised treatment approach to children with *C. burnetii* endocarditis.

## 1. Introduction

Q fever, first described in 1937 by Derrick [1], is caused by the obligate intracellular bacterium *Coxiella burnetii* [2]. In 2019, the reported incidence of Q fever in Europe/European Economic Area countries was 0.2 per 100,000 population [3]. Acute infection is often asymptomatic [4, 5]; however, a small proportion of cases progress to persistent localised infections, such as endocarditis, vascular infections, osteomyelitis, or lymphadenitis [6].

The management of persistent *C. burnetii* infections requires prolonged antimicrobial therapy [7]. Current recommendations for *C. burnetii* endocarditis advise a minimum treatment duration of 18–24 months with a combination of doxycycline and hydroxychloroquine [8–11]. The most commonly used criteria for defining serological cure are either a decrease of Immunoglobulin (Ig) G Phase I titres to < 1:800 or < 1:200 [8, 10], or recently, a fourfold decrease of Phase I IgG and IgA along with the complete disappearance of Phase II IgM, and the absence of clinical or biological signs of disease progression [7, 8]. However, the applicability of these criteria to paediatric patients remains uncertain, as current recommendations are based on adult data [8].

## 2. Case Presentation

A 6‐year‐old male patient presented to the emergency department with a 6‐week history of undulating fever and cough. His medical history was significant for a complex congenital heart disease with pulmonary atresia and ventricular septal defect (VSD), aortopulmonary collateral arteries and multiple surgical corrections, including VSD closure, unifocalisation and placement of a Contegra conduit in the pulmonary position. His most recent cardiac intervention, transapical implantation of a bovine prosthetic pulmonary valve (Melody), had been performed 6 months earlier. He had pulmonary hypertension, for which he was receiving oral sildenafil, and only his right lung lobe was perfused. Additionally, he had been born prematurely, had a ventriculoperitoneal shunt (implanted for posthaemorrhagic hydrocephalus), cerebral palsy and a global developmental delay with features of autism spectrum disorder.

His general practitioner had prescribed a 10‐day course of amoxicillin, followed by a 10‐day course of clarithromycin for suspected pneumonia. However, there was no sustained improvement. His family resided in a rural area near livestock farms but reported no direct contact with animals or recent travel abroad.

On clinical examination, the patient had low‐grade fever (37.8°C) and exhibited general fatigue but no signs of respiratory distress. A Grade‐4 holosystolic murmur and mild hepatosplenomegaly were unchanged from his prior cardiac outpatient assessment. Inflammatory markers were slightly elevated with a C‐reactive protein level of 28 mg/L and erythrocyte sedimentation rate of 19 mm/h. Testing for respiratory viruses was negative, and a chest X‐ray revealed no evidence of pneumonia. Blood cultures (two sets) remained sterile. Transthoracic echocardiography (TTE) showed no overt signs of endocarditis, or deterioration in cardiac function. Given the absence of specific findings, he was discharged without antimicrobial therapy.

Two weeks later, following an afebrile interval, the patient re‐presented to the outpatient clinic with fever and weight loss. Given the patient’s history, the diagnostic workup was expanded to include culture‐negative endocarditis. Serological testing revealed a *C. burnetii* Phase I IgG antibody titre of 1:65,536 (Table 1), confirming persistent *C. burnetii* infection. Culture and polymerase chain reaction testing for *C. burnetii* in blood remained negative.

**TABLE 1:** *Coxiella burnetii* serology during and after antimicrobial therapy.

	**Start of therapy (0 months)**	**End of therapy (40 months)**	**Serological follow-up (52 months)**	**Last serological follow-up (63 months)**

*C. burnetii* Phase I IgG (titer)	1:65,536	1:4096	1:1024	1:512
*C. burnetii* Phase II IgG (titer)	< 1:65,536	1:16,384	1:8192	1:4096
*C. burnetii* Phase I IgM (titer)	1:16,384	1:2048	1:1024	1:512
*C. burnetii* Phase II IgM (titer)	1:16,384	Not detected	Not detected	Not detected
*C. burnetii* Phase I IgA (titer)	1:160	Not detected	Not detected	Not detected
*C. burnetii* Phase II IgA (titer)	1:80	Not detected	Not detected	Not detected

**FIGURE 1:**
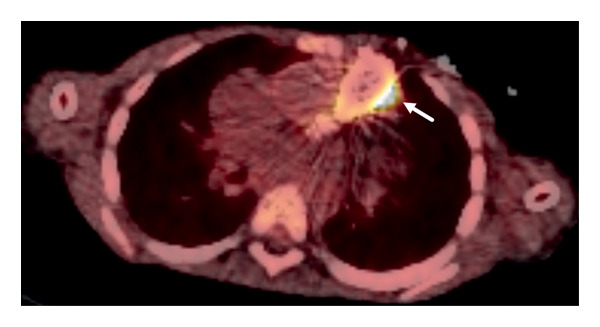
^18^F‐FDG PET scan of the thorax showing hypermetabolism lining the bovine pulmonary valve (white arrow).

As the site of *C. burnetii* infection could not be confirmed, and TTE was deemed insufficiently sensitive due to the patient’s underlying heart disease, a whole‐body 18‐fluorodeoxyglucose positron emission tomography (18F‐FDG PET) scan was performed. The scan revealed increased metabolic activity surrounding the right ventricle and the pulmonary conduit (Figure 1). Further diagnostic workup did not reveal any autoimmune phenomena.

Nine weeks after symptom onset, the patient was diagnosed with definite *C. burnetii* endocarditis based on the presence of 2 major and 2 minor criteria according to the Duke–International Society for Cardiovascular Infectious Diseases (ISCVID) infective endocarditis (IE) criteria [12, 13].

**FIGURE 2:**
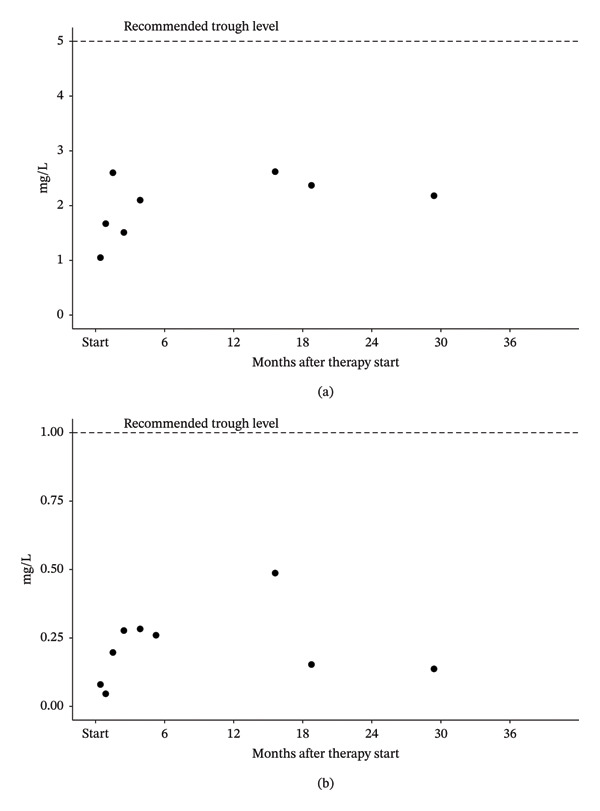
Trough levels of (a) doxycycline and (b) hydroxychloroquine during therapy, as measured by liquid chromatography tandem–mass spectrometry. Blood samples for measuring drug levels were taken 12–15 h after the last previous dose. Recommended trough level goals [8] are indicated with a dashed line.

Treatment for *C. burnetii* endocarditis was initiated with doxycycline at 2.2 mg/kg twice daily, and hydroxychloroquine at 5 mg/kg once daily. The patient’s general condition improved rapidly following treatment initiation, and after 2 weeks, his parents reported a return to normal physical activity within his individual limitations. In accordance with published recommendations [8], we aimed for doxycycline plasma concentrations of 5–10 mg/L and hydroxychloroquine levels of 0.8–1.2 mg/L. However, these target levels were not achieved with the initial dosing regimen. The doxycycline dose was progressively increased to a maximum of 5.8 mg/kg/day in two divided doses, and hydroxychloroquine to 11.7 mg/kg/day, within the first 3 months of therapy. Due to the unavailability of liquid formulations and the limited range of tablet strengths, the required doses were specifically compounded by the hospital pharmacy according to the patient’s weight. Despite these adjustments, plasma drug concentrations remained well below the target range (Figure 2). Pharmacodynamical modelling suggested that achieving target drug levels would require a threefold dose increase, posing a substantial risk of drug toxicity. Consequently, further dose escalation was deemed unfeasible.

The patient experienced phototoxicity in sun‐exposed areas, despite consistent sunscreen use. Additionally, when increasing the hydroxychloroquine dose, he started to vomit, which resolved after splitting the daily dose into two administrations. Monitoring for further adverse effects was complicated by his developmental delay, particularly regarding assessments for retinal toxicity, which were challenging to perform reliably.

During serological follow‐up, we documented a gradual decline in *C. burnetii* Phase I IgG antibody titres, with a fourfold reduction achieved after 6 months of therapy; additionally, Phase I IgA titres became negative after 9 months. Because of persistent documentation of *C. burnetii* IgM Phase II antibodies, we opted to extend therapy beyond 24 months. *C. burnetii* Phase II IgM titres turned negative after 36 months of therapy (Table 1). A follow‐up 18F‐FDG PET scan at 39 months of therapy demonstrated only minimal residual metabolic activity at the pulmonary conduit. Based on the clinical course, laboratory findings and imaging results, we deemed predefined criteria for stopping antimicrobial therapy to be met. After a shared decision‐making process with the parents, we discontinued antimicrobial therapy after 40 months.

At no point during follow‐up did the patient show clinical signs of active *C. burnetii* endocarditis, with a continuous decline of serological markers until the last measurement at 23 months after treatment cessation (Table 1). The Melody valve retained its function, and valve replacement was not required. The patient died 25 months after therapy end due to progressive heart failure associated with treatment‐resistant pulmonary hypertension.

## 3. Discussion

Paediatric *C. burnetii* endocarditis is rarely reported in the medical literature. Through a systematic literature search using Medline, Embase, Web of Science, and Scopus, we identified 40 individual case reports in patients younger than 18 years that have been published in the English medical literature until June 2025 (Supporting File (available here)). We identified 34 (85%) cases with definite and six (15%) possible *C. burnetii* endocarditis according to the Duke‐ISCVID IE criteria [12]. However, in 5 cases (2 definite and 3 possible endocarditis) microbiological criteria for persistent *C. burnetii* infection were not fulfilled (Supporting File (available here)). Thirty‐five (88%) patients had CHD, 29 (73%) with a prosthetic valve at the time of endocarditis (Supporting File (available here)), underscoring that patients with congenital heart disease, especially those with prosthetic valves, are at an increased risk to develop endocarditis after acute *C. burnetii* infection [9, 14].

Few reports provided information on treatment duration and follow‐up underscoring the lack of clinical evidence and guidance for optimal management of paediatric patients with *C. burnetii* endocarditis.

The preferred treatment regimen for *C. burnetii* endocarditis consists of doxycycline and hydroxychloroquine [8]. Of the published paediatric cases, 28 (70%) patients received this combination therapy (Supporting File (available here)). In children, the recommended doxycycline dose is 2.2 mg/kg every 12 h (maximum daily dose: 200 mg), while hydroxychloroquine is administered at 5 mg/kg every 24 h (maximum daily dose: 600 mg, in three divided doses). However, despite progressively increasing drug doses, we were unable to achieve recommended target trough levels [10, 15]. Given the favourable clinical course, increasing adverse effects and pharmacological modelling indicating that target drug concentrations could only be reached with potentially toxic drug doses, we decided to maintain therapy at the highest tolerable drug doses.

There is limited evidence on the long‐term use of doxycycline in children. While recent studies suggest that short‐term doxycycline therapy does not cause dental staining [16, 17], there are little data on prolonged use in paediatric patients [8]. The adverse effects observed in our patient—photosensitivity, nausea and vomiting—are well documented for both doxycycline [18] and hydroxychloroquine [19]. Although hydroxychloroquine has been safely used long‐term in children with systemic lupus erythematosus, monitoring for retinal toxicity is required [20, 21]. Notably, the patient’s developmental disorder significantly limited our ability to perform regular ophthalmological screening for retinal toxicity.

For patients with prosthetic valve *C. burnetii* endocarditis, the minimum recommended treatment duration is 24 months because of the high risk of infection relapse with shorter treatment durations [7, 8]. In the published paediatric cases, therapy durations ranged from 7 to 32 months, with the most common choices being 18 (11, 28%) or 24 (10, 25%) months (Supporting File (available here)). Previously, a Phase‐I IgG titre < 1:800 was considered a serological marker of cure required before discontinuing therapy for persistent *C. burnetii* infection [10]. Recent recommendations suggest that treatment should be extended beyond the minimum duration if Phase I IgG titre fails to decrease fourfold or if IgA and IgM antibodies remain detectable, as these findings have been associated with higher mortality [7, 8]. Some experts challenge the clinical relevance of serological markers for defining treatment duration [22]. In our patient, a fourfold reduction in Phase I IgG antibody titres occurred within the first 6 months of therapy, yet Phase II IgM antibodies remained detectable for 36 months. Given the high surgical risk in our patient, we decided to discontinue antimicrobial therapy only once predefined clinical, serological and imaging criteria were met. With this approach, Melody valve function was preserved and cardiac surgery was avoided. Of the published paediatric cases, 23 (58%) patients required a surgical intervention (Supporting File (available here)).

In 23 (58%) of the reported paediatric cases, cardiac surgery was performed (Supporting File (available here)), which is comparable to data from adult studies [7, 23], with one site reporting a secular trend to less surgery [7]. Indications for early cardiac surgery are the same as for other forms of IE, heart failure following valve dysfunction and local or distant complications of IE, with late surgery usually indicated because of valve dysfunction [24]. While surgery has been reported to be of critical importance in the management of *C. burnetii* vascular infections [25], data for *C. burnetii* endocarditis are less clear [7]. In our patient, cardiac surgery was not performed, given the favourable clinical course without complications, and the difficult anatomical situation.

The patient died 25 months after discontinuation of antimicrobial therapy due to heart failure associated with treatment‐resistant pulmonary hypertension. At no point did he show clinical or serological signs of active *C. burnetii* endocarditis; however, we acknowledge the fact that we are unable to provide microbiological proof as the parents refused an autopsy.

In conclusion, this case highlights the challenges in defining optimal treatment strategies for paediatric *C. burnetii* endocarditis, given the scarcity of clinical evidence. Our experience suggests that strict adherence to adult‐based treatment recommendations may not be feasible in all children, necessitating individualised treatment approaches.

## Funding

Open access publishing was facilitated by Inselspital Universitatsspital Bern, as part of the Wiley–Inselspital Universitatsspital Bern agreement via the Consortium Of Swiss Academic Libraries.

## Disclosure

This work was performed as part of the employment of the authors at the Inselspital, Bern University Hospital, University of Bern, Switzerland.

## Consent

Written consent for the authorisation for the publication of the case report was obtained from the patient’s family. A blank copy of the consent form for the case report was provided to the journal.

## Conflicts of Interest

The authors declare no conflicts of interest.

## Supporting Information

Additional supporting information can be found online in the Supporting Information section.

## Supporting information


**Supporting Information** Supporting Table 1: Previously published *Coxiella burnetii* endocarditis cases in children and adolescents. CARE Checklist of information to include when writing a case report.

## Data Availability

Data sharing is not applicable to this article as no datasets were generated or analysed during the current study.

## References

[1] Derrick E. H. , Q Fever, a New Fever Entity: Clinical Features, Diagnosis and Laboratory Investigation, Reviews of Infectious Diseases. (1983) 5, no. 4, 790–800, 10.1093/clinids/5.4.790. 6622891

[2] McCaul T. F. and Williams J. C. , Developmental Cycle of *Coxiella Burnetii*: Structure and Morphogenesis of Vegetative and Sporogenic Differentiations, Journal of Bacteriology. (1981) 147, no. 3, 1063–1076, 10.1128/jb.147.3.1063-1076.1981. 7275931 PMC216147

[3] Q fever—Annual Epidemiological Report for 2019 , Annual Epidemiological Report, 2021, European Centre for Disease Prevention and Control, 1–6.

[4] Dupuis G. , Petite J. , Péter O. , and Vouilloz M. , An Important Outbreak of Human Q Fever in a Swiss Alpine Valley, International Journal of Epidemiology. (1987) 16, no. 2, 282–287, 10.1093/ije/16.2.282, 2-s2.0-0023182157. 3301708

[5] Maurin M. and Raoult D. , Q Fever, Clinical Microbiology Reviews. (1999) 12, no. 4, 518–553, 10.1128/cmr.12.4.518. 10515901 PMC88923

[6] Eldin C. , Mélenotte C. , Mediannikov O. et al., From Q Fever to *Coxiella burnetii* Infection: A Paradigm Change, Clinical Microbiology Reviews. (2017) 30, no. 1, 115–190, 10.1128/cmr.00045-16, 2-s2.0-85007162599. 27856520 PMC5217791

[7] Million M. , Thuny F. , Richet H. , and Raoult D. , Long-Term Outcome of Q Fever Endocarditis: A 26-Year Personal Survey, Lancet Infectious Diseases. (2010) 10, no. 8, 527–535, 10.1016/s1473-3099(10)70135-3, 2-s2.0-77955096262. 20637694

[8] Anderson A. , Bijlmer H. , Fournier P. E. et al., Diagnosis and Management of Q Fever—United States, 2013: Recommendations From CDC and the Q Fever Working Group, Morbidity and Mortality Weekly Report Recommendations and Reports. (2013) 62, no. RR-03, 1–30. 23535757

[9] Fenollar F. , Fournier P. , Carrieri M. , Habib G. , Messana T. , and Raoult D. , Risks Factors and Prevention of Q Fever Endocarditis, Clinical Infectious Diseases. (2001) 33, no. 3, 312–316, 10.1086/321889, 2-s2.0-0034928070. 11438895

[10] Raoult D. , Houpikian P. , Dupont H. T. , Riss J. M. , Arditi-Djiane J. , and Brouqui P. , Treatment of Q Fever Endocarditis: Comparison of 2 Regimens Containing Doxycycline and Ofloxacin or Hydroxychloroquine, Archives of Internal Medicine. (1999) 159, no. 2, 167–173, 10.1001/archinte.159.2.167, 2-s2.0-0033601740. 9927100

[11] Sobradillo V. , Zalacain R. , Capelastegui A. , Uresandi F. , and Corral J. , Antibiotic Treatment in Pneumonia due to Q Fever, Thorax. (1992) 47, no. 4, 276–278, 10.1136/thx.47.4.276, 2-s2.0-0026529375. 1585291 PMC463691

[12] Fowler V. G. , Durack D. T. , Selton-Suty C. et al., The 2023 Duke-International Society for Cardiovascular Infectious Diseases Criteria for Infective Endocarditis: Updating the Modified Duke Criteria, Clinical Infectious Diseases. (2023) 77, no. 4, 518–526, 10.1093/cid/ciad271. 37138445 PMC10681650

[13] Li J. S. , Sexton D. J. , Mick N. et al., Proposed Modifications to the Duke Criteria for the Diagnosis of Infective Endocarditis, Clinical Infectious Diseases. (2000) 30, no. 4, 633–638, 10.1086/313753, 2-s2.0-0033796633. 10770721

[14] Puyana-Rodriguez J. M. , Guida-Piqueras M. , Navas-Elorza E. et al., Endocarditis due to *Coxiella burnetii* in Congenital Heart Disease Patients: A Clinical Case Series and Diagnostic Considerations, Revista Española de Quimioterapia. (2025) 38, no. 6, 527–530, 10.37201/req/074.2025. 41342595 PMC12707431

[15] Rolain J. M. , Mallet M. N. , and Raoult D. , Correlation Between Serum Doxycycline Concentrations and Serologic Evolution in Patients With *Coxiella burnetii* Endocarditis, Journal of Infectious Diseases. (2003) 188, no. 9, 1322–1325, 10.1086/379082, 2-s2.0-0242721038. 14593588

[16] Gaillard T. , Briolant S. , Madamet M. , and Pradines B. , The End of a Dogma: the Safety of Doxycycline Use in Young Children for Malaria Treatment, Malaria Journal. (2017) 16, no. 1, 10.1186/s12936-017-1797-9, 2-s2.0-85018523831. PMC5390373 28407772

[17] Wormser G. P. , Wormser R. P. , Strle F. , Myers R. , and Cunha B. A. , How Safe is Doxycycline for Young Children or for Pregnant or Breastfeeding Women?, Diagnostic Microbiology and Infectious Disease. (2019) 93, no. 3, 238–242, 10.1016/j.diagmicrobio.2018.09.015, 2-s2.0-85056275911. 30442509

[18] Blakely K. M. , Drucker A. M. , and Rosen C. F. , Drug-Induced Photosensitivity-An Update: Culprit Drugs, Prevention and Management, Drug Safety. (2019) 42, no. 7, 827–847, 10.1007/s40264-019-00806-5, 2-s2.0-85063207229. 30888626

[19] Fox J. N. , Klapman M. H. , and Rowe L. , Lupus Profundus in Children: Treatment With Hydroxychloroquine, Journal of the American Academy of Dermatology. (1987) 16, no. 4, 839–844, 10.1016/s0190-9622(87)70110-8, 2-s2.0-0023150640. 3571546

[20] Marmor M. F. , Kellner U. , Lai T. Y. , Melles R. B. , and Mieler W. F. , Recommendations on Screening for Chloroquine and Hydroxychloroquine Retinopathy (2016 Revision), Ophthalmology. (2016) 123, no. 6, 1386–1394, 10.1016/j.ophtha.2016.01.058, 2-s2.0-84961161700. 26992838

[21] Ponticelli C. and Moroni G. , Hydroxychloroquine in Systemic Lupus Erythematosus (SLE), Expert Opinion on Drug Safety. (2017) 16, no. 3, 411–419, 10.1080/14740338.2017.1269168, 2-s2.0-85006142620. 27927040

[22] Buijs S. B. , van Roeden S. E. , van Werkhoven C. H. et al., The Prognostic Value of Serological Titres for Clinical Outcomes During Treatment and Follow-Up of Patients With Chronic Q Fever, Clinical Microbiology and Infections. (2021) 27, no. 9, 1273–1278, 10.1016/j.cmi.2021.03.016. 33813120

[23] Kampschreur L. M. , Delsing C. E. , Groenwold R. H. H. et al., Chronic Q Fever in the Netherlands 5 Years After the Start of the Q Fever Epidemic: Results From the Dutch Chronic Q Fever Database, Journal of Clinical Microbiology. (2014) 52, no. 5, 1637–1643, 10.1128/jcm.03221-13, 2-s2.0-84899522116. 24599987 PMC3993626

[24] Delgado V. , Ajmone Marsan N. , de Waha S. et al., 2023 ESC Guidelines for the Management of Endocarditis, European Heart Journal. (2023) 44, no. 39, 3948–4042, 10.1093/eurheartj/ehad193. 37622656

[25] Botelho-Nevers E. , Fournier P. E. , Richet H. et al., *Coxiella burnetii* Infection of Aortic Aneurysms or Vascular Grafts: Report of 30 New Cases and Evaluation of Outcome, European Journal of Clinical Microbiology & Infectious Diseases. (2007) 26, no. 9, 635–640, 10.1007/s10096-007-0357-6, 2-s2.0-34548119165. 17629755

