# Supplementary material for: Management of Coxiella burnetii Endocarditis in a Child With Congenital Heart Disease
Source: Case Rep Pediatr. 2026 May 28;2026:8653626. doi: 10.1155/crpe/8653626 (PMC13217158; doi:10.1155/crpe/8653626)
Supplement: Supplementary file 1 — Supporting Information Supporting Table 1: Previously published Coxiella burnetii endocarditis cases in children and adolescents. CARE Checklist of information to include when writing a case report. [file CRPE-2026-8653626-s001.pdf]

Supplementary material

Supplement to:
Management of Coxiella burnetii endocarditis in a child with congenital heart disease

Emilija Zimnickaite[1], Andrea Dulcey[1], Christoph Aebi[1,2], Andrea Duppenhaler[1,2], Nina Schöbi[1,2], Fabienne Stoller[1,3], Florian Arndt[1,3], Philipp KA Agyeman[1,2]

[1] Department of Pediatrics, Inselspital, Bern University Hospital, University of Bern, Switzerland
[2] Division of Pediatric Infectious Disease, Department of Pediatrics, Inselspital, Bern University Hospital, University of Bern, Switzerland
[3] Department of Cardiology, Pediatric Cardiology, Center for Congenital Heart Disease, Bern University Hospital, University of Bern, Switzerland

Table of content

Supplemental Table 1:... 2
References.. 5
Care Checklist... 7

Supplementary Table 1: Previously published *Coxiella burnetii* endocarditis cases in children and adolescents

Authors (Year)	Sex (Age in years)	Congenital heart disease / prosthetic valve	Endocarditis diagnosis#	Surgery	Antibiotics (therapy specifics or reason for change)	Treatment duration	Outcome
Kristinsson and Bentall (1967) [1]	Male (15)	No / No	Definite	Yes	TCY 1000 mg/d	N/A	No follow-up
Jones and Pitcher (1980) [2]	Male (6)	Yes / No	Possible¶	No	SXT§ & DOX§	N/A	No follow-up
Laufer et al (1986) [3]	Female (3.5)	Yes / No	Possible¶	No	DOX 60mg/d	N/A	No follow-up
	Female (4)	Yes / No	Definite	Yes	TCY§	N/A	No follow-up
Beauford-Krol and Storm (1987) [4]	Male (7)	No / No	Possible¶	No	DOX§	7 months	Cured
Lupoglazoff et al (1997) [5]	Female (8)	Yes / No	Definite	Yes	DOX 200 mg/d & HCQ 600 mg/d	Planned for 24 months	No follow-up
Al-Hajjar et al (1997) [6]	Male (11)	Yes / No	Definite	Yes	DOX 200 mg/d, switch to TCY 750 mg/d & RIF 250 mg/d	6 weeks<br>N/A<br>N/A	No follow-up
Sahagun Sanchez et al (1998) [7]	Female (10)	Yes / No	Definite	Yes	DOX§	N/A	No follow-up
Rovery et al (2009) [8]	Female (16)	No / No	Possible	Yes	DOX 200 mg/d & HCQ 600 mg/d	18 months	Cured
Mogollon et al (2011) [9]	Female (16)	N/A / No	Definite	Yes	DOX§ & HCQ§	11 months	Died
	Male (11)[10]*	Yes / Yes	Definite	Yes	DOX§	18 months	Cured*
Stefanidis et al (2011) [11]	Male (12)	Yes / Yes	Definite	Yes	DOX 200mg/d & HCQ 600 mg/d	Planned for 18 months	No follow-up
Ceylan et al (2013) [12]	Female (6)	Yes / Yes	Definite	No	DOX 4.4 mg/kg/d & HCQ 7 mg/kg/d	Planned for 18 months	No follow-up
Tasher et al (2012) [13]	Female (11)	Yes / Yes	Definite	Yes	DOX§ & HCQ§	Planned for 24 months	No follow-up
Eberhardt et al (2013) [14]	Male (14)	Yes / Yes	Definite	Yes	DOX§ & HCQ§	Planned for 18 months	No follow-up
Angelakis et al (2014) [15]	Male (13)	Yes / Yes	Definite	No	DOX 200mg/d & HCQ 600 mg/d	18 months	Cured

Abu Rmeileh et al (2015) [16]	Female (14)	No / No	Definite	No	DOX[§] & HCQ[§]	N/A	No follow-up
Briggs et al (2016) [17]	Male (13)	Yes / Yes	Definite	Yes	DOX 200mg/d & HCQ 400mg/d	Planned for 18 months	No follow-up
Maor et al (2016) [18]	Female (11)	Yes / Yes	Possible	Yes	DOX 200mg/d & HCQ 600 mg/d	Planned for 24 months	No follow-up
Alhadhoud et al (2018) [19]	Male (15)	Yes / Yes	Definite	Yes	DOX[§] & HCQ[§] & INH 300mg/d	N/A for DOX and HCQ, INH stopped after 3 months	No follow-up
	Female (12)	Yes / Yes	Definite	Yes	DOX[§] (AE) & HCQ[§] (AE), switch to MFX[§] & RIF[§]	Planned for 24 months	No follow-up
	Female (3)	Yes / Yes	Definite	Yes	DOX[§] & HCQ[§]	N/A	No follow-up
Sachs et al (2018) [20]	Female (5.5)	Yes / Yes	Definite	No	DOX[§] & HCQ[§]	36 months	Cured
	Male (17)	Yes / Yes	Possible	No	DOX[§] & CIP[§]	N/A	Cured
	Female (11)	Yes / Yes	Definite	Yes	DOX[§] & HCQ[§]	6 months	Cured
	Female (16)	Yes / Yes	Definite	No	DOX[§] & HCQ[§]	18 months	Cured
	Female (11)	Yes / Yes	Definite	No	DOX[§] & HCQ[§]	18 months	Cured
Kagan et al (2019) [21]	Male (2.5)	Yes / Yes	Definite	Yes	DOX 4 mg/kg/d & RIF 20 mg/kg/d	Planned for 24 months	No follow-up
Alzahrani et al (2019) [22]	Male (3)	Yes / Yes	Definite	No	DOX[§] (initial monotherapy) & CIP[§]	Planned for 18 months	No follow-up
Elzein et al (2019) [23]	N/A (12)	Yes / Yes	Definite	Yes	DOX[§] & HCQ[§]	24 Months	Cured
	N/A (12)	Yes / Yes	Definite[¶]	No	DOX[§] & HCQ[§]	24 Months	Cured
	N/A (13)	Yes / Yes	Definite	Yes	DOX[§]	10 months	Died
	N/A (13)	Yes / Yes	Definite	No	DOX[§] & HCQ[§]	24 Months	No follow-up
	N/A (12)	Yes / Yes	Definite	No	DOX[§] & HCQ[§]	24 Months	No follow-up
	N/A (14)	Yes / Yes	Definite	Yes	DOX[§] & HCQ[§]	24 Months	Cured
	N/A (17)	Yes / Yes	Definite	No	DOX[§] & HCQ[§]	N/A	No follow-up
	N/A (14)	Yes / Yes	Definite	No	DOX[§] & HCQ[§]	N/A	No follow-up

Al-Araimi et al (2020) [24]	Female (11)	Yes / Yes	Definite	No	DOX§ & HCQ§	32 months	Cured
Huguet et al (2023) [25]	Male (8)	Yes / Yes	Definite	Yes	DOX 4.4 mg/kg/d & HCQ 200 mg/d (AE) & SXT 8mg/kg/d	19 months<br>2 weeks<br>19 months	Cured
Hijazi et al (2023) [26]	Male (15)	Yes / Yes	Definite¶	Yes	DOX 200mg/d & HCQ§ (AE) & CIP 1000mg/d	N/A<br>8 weeks<br>N/A	No follow-up

AE, adverse effects; CIP, ciprofloxacin; DOX, Doxycycline; HCQ, hydroxychloroquine; INH, isoniazid; MXF, moxifloxacin; N/A, not available; RIF, rifampin; SXT, Co-trimoxazole; TCY, Tetracycline

According to the 2023 Duke-International Society for Cardiovascular Infectious Diseases (ISCVID) criteria for infective endocarditis [27]

* Case has been reported previously by Tellez et al. [10] with sufficient information to be categorised as definite *C. burnetii* endocarditis (confirmed by the authors)

§ drug dose not specified

¶ Case meeting the 2023 Duke-International Society for Cardiovascular Infectious Diseases (ISCVID) criteria for infective endocarditis [27], but not meeting the *Coxiella burnetii* evidence level

References

1. Kristinsson, A. and H.H. Bentall, *Medical and surgical treatment of Q-fever endocarditis.* Lancet, 1967. **2**(7518): p. 693-7.
2. Jones, R.W. and D.W. Pitcher, *Q fever endocarditis in a 6-year-old child.* Arch Dis Child, 1980. **55**(4): p. 312-5.
3. Laufer, D., et al., *Chronic Q fever endocarditis with massive splenomegaly in childhood.* J Pediatr, 1986. **108**(4): p. 535-9.
4. Beaufort-Krol, G.C. and C.J. Storm, *Chronic Q fever endocarditis.* J Pediatr, 1987. **110**(2): p. 330-1.
5. Lupoglazoff, J.M., et al., *Q fever tricuspid valve endocarditis.* Arch Dis Child, 1997. **77**(5): p. 448-9.
6. al-Hajjar, S., et al., *Coxiella burnetii endocarditis in a child.* Pediatr Infect Dis J, 1997. **16**(9): p. 911-3.
7. Sahagun Sanchez, G., et al., *[Coxiella burnetii endocarditis. A report of the first case diagnosed in Mexico].* Arch Inst Cardiol Mex, 1998. **68**(4): p. 322-7.
8. Rovery, C., et al., *Coinfection with Coxiella burnetii in infectious endocarditis.* Clin Microbiol Infect, 2009. **15 Suppl 2**: p. 190-1.
9. Mogollon, M.V., et al., *Q fever endocarditis in Spain. Clinical characteristics and outcome.* Enferm Infecc Microbiol Clin, 2011. **29**(2): p. 109-16.
10. Tellez, A., et al., *Q fever in Spain: acute and chronic cases, 1981-1985.* Rev Infect Dis, 1988. **10**(1): p. 198-202.
11. Stefanidis, C., et al., *Endocarditis of bovine jugular vein conduit due to Q fever.* Ann Thorac Surg, 2011. **91**(6): p. 1990-2.
12. Ceylan, O., et al., *Coxiella burnetii endocarditis presented with fever of unknown origin in a child with operated congenital heart disease.* Turk Pediatri Arsivi, 2013. **48**(4): p. 339-341.
13. Tasher, D., et al., *Coxiella burnetii infection of a bovine jugular vein conduit in a child.* Pediatr Cardiol, 2012. **33**(5): p. 831-3.
14. Eberhardt, C.S., C. Tissot, and K.M. Posfay-Barbe, *Don't forget the C in endoCarditis-a case of chronic Coxiella burnetii infection in an adolescent cardiac patient with a pulmonary valved conduit xenograft.* Swiss Medical Weekly, 2013. **143**: p. 42S.
15. Angelakis, E., et al., *Q fever endocarditis and new Coxiella burnetii genotype, Saudi Arabia.* Emerg Infect Dis, 2014. **20**(4): p. 726-8.
16. Abu Rmeileh, A., et al., *Familial Q fever clustering with variable manifestations imitating infectious and autoimmune disease.* Clin Microbiol Infect, 2015. **21**(5): p. 459-63.
17. Biggs, H.M., et al., *Coxiella burnetii Infection in a Community Operating a Large-Scale Cow and Goat Dairy, Missouri, 2013.* Am J Trop Med Hyg, 2016. **94**(3): p. 525-31.
18. Maor, Y., et al., *Coxiella burnetii Endocarditis and Aortic Vascular Graft Infection: An Underrecognized Disease.* Ann Thorac Surg, 2016. **101**(1): p. 141-5.
19. Alhadhoud, S.A., M.T. Vel, and M. Al Qbandi, *Q fever endocarditis after right ventricle to pulmonary artery conduit insertion: Case series and review of the literature.* Ann Pediatr Cardiol, 2018. **11**(1): p. 60-63.
20. Sachs, N., et al., *Chronic Q Fever Infections in Israeli Children: A 25-year Nationwide Study.* Pediatr Infect Dis J, 2018. **37**(3): p. 212-217.
21. Kagan, S., et al., *Q Fever and Kingella kingae Endocarditis in a Toddler: A Rare Coinfection Case.* Pediatr Infect Dis J, 2019. **38**(12): p. e336-e337.
22. Alzahrani, A., et al., *Q Fever Endocarditis in a Saudi Child: A Case Report and Literature Review.* Cureus, 2019. **11**(12): p. e6322.
23. Elzein, F.E., et al., *Ten-year experience of Q fever endocarditis in a tertiary cardiac center in Saudi Arabia.* Int J Infect Dis, 2019. **88**: p. 21-26.

24. Al-Araimi, H.A., et al., *Chronic Q Fever Endocarditis in an Omani Child: The First Pediatric Case Report from Oman.* Oman Med J, 2020. **35**(5): p. e180.
25. Huguet, B., et al., *Prolonged Fever in a Child With Congenital Heart Disease and a Prosthetic Valve.* Pediatric Infectious Disease Journal, 2023. **42**(4): p. 350-352.
26. Hijazi, R., et al., *Coxiella burnetii-Associated Endocarditis in a Saudi Adolescent: A Rare Encounter.* Cureus, 2023. **15**(12): p. e50470.
27. Fowler, V.G., et al., *The 2023 Duke-International Society for Cardiovascular Infectious Diseases Criteria for Infective Endocarditis: Updating the Modified Duke Criteria.* Clin Infect Dis, 2023. **77**(4): p. 518-526.

Care Checklist

CARE case report guidelines

CARE Checklist of information to include when writing a case report

(cc) BY-NC-ND

Topic	Item	Checklist item description	Reported on Line
Title	1	The diagnosis or intervention of primary focus followed by the words "case report"	1-3
Key Words	2	2 to 5 key words that identify diagnoses or interventions in this case report, including "case report"	26
Abstract (no references)	3a	Introduction: What is unique about this case and what does it add to the scientific literature?	33-41
	3b	Main symptoms and/or important clinical findings	29
	3c	The main diagnoses, therapeutic interventions, and outcomes	30-40
	3d	Conclusion—What is the main "take-away" lesson(s) from this case?	40-41
Introduction	4	One or two paragraphs summarizing why this case is unique (may include references)	43-58
Patient Information	5a	De-identified patient specific information.	60-70
	5b	Primary concerns and symptoms of the patient.	60-61, 84-85
	5c	Medical, family, and psycho-social history including relevant genetic information	62-70
	5d	Relevant past interventions with outcomes	71-73
Clinical Findings	6	Describe significant physical examination (PE) and important clinical findings.	75-77
Timeline	7	Historical and current information from this episode of care organized as a timeline	Not applicable
Diagnostic Assessment	8a	Diagnostic testing (such as PE, laboratory testing, imaging, surveys).	78-82, 86-89, 91-95
	8b	Diagnostic challenges (such as access to testing, financial, or cultural)	118 - 120
	8c	Diagnosis (including other diagnoses considered)	96-99
	8d	Prognosis (such as staging in oncology) where applicable	Not applicable
Therapeutic Intervention	9a	Types of therapeutic intervention (such as pharmacologic, surgical, preventive, self-care)	100-101
	9b	Administration of therapeutic intervention (such as dosage, strength, duration)	100-101, 107-108, 129
	9c	Changes in therapeutic intervention (with rationale)	104-115, 116-118
Follow-up and Outcomes	10a	Clinician and patient-assessed outcomes (if available)	101-103, 130-134
	10b	Important follow-up diagnostic and other test results	121-129
	10c	Intervention adherence and tolerability (How was this assessed?)	104-106, 116-120, 273
	10d	Adverse and unanticipated events	116-118
Discussion	11a	A scientific discussion of the strengths AND limitations associated with this case report	136-185
	11b	Discussion of the relevant medical literature with references.	136-185
	11c	The scientific rationale for any conclusions (including assessment of possible causes)	136-185
	11d	The primary "take-away" lessons of this case report (without references) in a one paragraph conclusion	187-191
Patient Perspective	12	The patient should share their perspective in one to two paragraphs on the treatment(s) they received	Not appliable
Informed Consent	13	Did the patient give informed consent? Please provide if requested	Yes ☒ No ☐
